# Cutaneous Lesions in Iranian Neonates and Their Relationships with Maternal-Neonatal Factors: A Prospective Cross-Sectional Study

**DOI:** 10.1155/2020/8410165

**Published:** 2020-05-05

**Authors:** Hossein Firouzi, Iman Jalalimehr, Zahra Ostadi, Siavash Rahimi

**Affiliations:** ^1^Department of Pediatrics, Ramsar Campus, Mazandaran University of Medical Sciences, Sari, Iran; ^2^Department of Pediatrics, Shahid Rajaee Hospital, Mazandaran University of Medical Sciences, Tonekabon, Iran

## Abstract

Cutaneous lesions are common in the neonatal period and mostly physiological, transient, and self-limited; uncommonly, they are pathological and require treatment and cooperation between neonatologists and dermatologists. Particular conditions, like prematurity, can influence the onset, type, and evolution of cutaneous manifestations. Of the several articles in the literature about skin findings in newborns, only a few were performed in Iran. We aimed to investigate dermatological findings in a sample of neonates within the first three days of life and to evaluate the association between skin lesions and neonatal- or maternal-related variables. A total of 1202 newborns, hospitalized in the Department of Pediatrics of Imam Sajjad Hospital of Ramsar and Shahid Rajaee Hospital of Tonekabon, Iran, for two years, were examined. All skin findings were recorded, and information on neonatal and maternal variables was collected and analyzed to detect statistically significant associations. Skin lesions were present in 958 newborns (79.8%). The prevalence of milia, erythema toxicum, salmon patch, and Mongolian spots were 45.2%, 43%, 37.3%, and 37%, respectively. Natural vaginal delivery, use of medication, term gestation, and maternal disease were associated with a higher incidence of cutaneous lesions in neonates. Milia, erythema toxicum, Mongolian spots, and genital hyperpigmentation were seen more frequently in the male gender. Conversely, skin desquamation was seen more frequently in females. Among maternal diseases, gestational diabetes mellitus, urinary tract infection, preeclampsia, hypertension, psychiatric disorders, and uterine infection were associated with a higher prevalence of cutaneous lesions. Neonatal cutaneous lesions are a common source of concern in parents and inexperienced physicians. Therefore, prompt recognition of neonatal cutaneous lesions is essential in order to avoid unnecessary diagnostic and therapeutic procedures.

## 1. Introduction

The skin of the infant differs from that of the adult, in that it is thinner and delicate, has weaker intercellular attachments, and produces less sweat and sebaceous gland secretions and it is more susceptible to several infections. The neonatal period is defined as the first 28 days of extrauterine life. The neonatal integumentary system offers physical protection, fluid balance regulation, immunosurveillance, and heat preservation, therefore playing a vital role in the newborn's transition from an aqueous to an air-dominant environment [1, 2]. In this period, dermatological findings are prevalent and can differ significantly from physiological and self-limited lesions caused by the developmental process of the integumentary system to severe pathological issues, which requires interventions and collaboration between specialists [3]. Therefore, early recognition is essential to differentiate benign lesions from more serious disorders and to provide proper counseling to parents. Several fetal, maternal, and environmental factors, such as prematurity, congenital infections, ethnicity, and maternal drug use, can influence the onset, type, and evolution of cutaneous lesions [3–6]. Previous studies have been performed regarding cutaneous lesions in neonates and their association with fetal conditions and maternal factors, yet only a few of these studies were performed in a diverse population of Iran [7–9]. Our study aimed to investigate dermatological findings in a sample of Iranian neonates in the north of Iran within the first 72 hours of their extrauterine life and to evaluate the potential influence of several neonatal-, parental-, or pregnancy-related variables in the onset of dermatological findings.

## 2. Materials and Methods

This multicenter hospital-based study was conducted at two major general hospitals in the west of Mazandaran province, Iran. In this cross-sectional study, 1202 consecutive newborns were evaluated for two years (2017–2019) at Imam Sajjad hospital of Ramsar and Shahid Rajaee hospital of Tonekabon, Mazandaran University of Medical Sciences. All newborns in regular postnatal wards who were no more than 72 hours of age were included. The study protocol was approved by the ethical committee of Mazandaran University of Medical Sciences before enrolling any participants. Also, prior to enrollment, written informed consent was obtained from participants' parents. A single dermatologist and neonatologist examined all patients within 72 hours of life. Entire skin surface, including palms, soles, genitalia, and scalp, was carefully examined, and cutaneous lesions were diagnosed based on clinical and morphologic features; no skin biopsy was performed. In the event of diagnoses requiring observation or therapy, further management was shared with the parents. Data regarding the neonatal history of the participating neonates (gestational age, maternal comorbidities, mode of delivery, birth weight, maternal age, parity, and maternal drug use) were obtained from the official medical profiles and interviews by a neonatologist.

Based on 35% prevalence, the confidence level of 99%, the margin of error 5%, and population size of 100,000 samples were calculated to be 1202 with the following equations:

(1) $$\begin{array}{c} X=\frac{Z_{\left(\alpha /2\right)}^{2}*p*q}{{\text{MOE}}^{2}}, \end{array}$$

(2) $$\begin{array}{c} n=2\left(N*\frac{X}{X+N-1}\right). \end{array}$$

At the end of the study, the collected data were analyzed using SPSS v22.0 (IBM Corp., Armonk, N.Y, USA). Descriptive statistical methods—means, standard deviations (SDs), and frequencies—were used to analyze the variables. Comparisons of variables were performed using the chi-square test, Fischer exact test, and independent *t*-test. *P* values of less than 0.05 were considered to indicate statistical significance.

## 3. Results

In this study, 1200 newborns were evaluated. Due to missing documents, 2 patients were excluded. Maternal and neonatal characteristics and their association with cutaneous lesions are mentioned in Tables 1 and 2, respectively. 958 (79.8%) of newborns had cutaneous lesions. The prevalence of different maternal diseases and their association with cutaneous lesion are shown in Table 3. The prevalence of cutaneous lesions based on genders is presented in Table 4.

## 4. Discussion

Although the majority of cutaneous conditions in the newborns are benign, they are the most encountered condition in general neonatal pediatric practice [10]. Thus, it is essential to differentiate physiologic skin lesions from pathologic ones to avoid unnecessary diagnostic or therapeutic procedures and parents' concerns. This study was a clinical and statistical survey of neonatal cutaneous conditions during the first three days of extrauterine life in Iranian neonates. In this study, 79.8% of newborns had at least one cutaneous lesion. The four most common cutaneous conditions found in this study were milia (45.2%), erythema toxicum (43%), salmon patch (37.3%), and Mongolian spots (37%).

There are few reports from Iran, which are overviewed here. Karegar Maher et al. reported salmon patch and Mongolian spots frequency in the Northwest of Iran [7]. According to the results, the frequency rates of Mongolian spots and salmon patch among 1000 newborns were found to be 32.3% and 14.5%, respectively. Maternal age was the only factor that showed a statistically significant association with the salmon patch. In addition, upper eyelid and sacral regions were found to be the most common site of the salmon patch and Mongolian spots, respectively [7].

Shajari et al. evaluated the incidence of different birthmarks in 503 neonates in Tehran, Iran [9]. Epstein's pearls, Mongolian spots, erythema toxicum, sucking blisters, salmon patch, milia, petechial, and mottling were found in 88.27%, 81%, 54%, 52.1%, 52%, 46%, 0.08%, and 0.06% of neonates, respectively. Petechia was reported more frequently in vaginal delivery and neonates with higher birth weight. Mottling was more common in premature and low-birth-weight babies [9].

Khoshnevisasl et al. evaluated the incidence of birthmarks in neonates born in Zanjan, Iran [8]. Among examined 500 neonates, 95.6% had at least one lesion. The most common skin lesions were Epstein's pearl (60.4%) and Mongolian spots (56%), respectively. Mongolian spots were significantly associated with gestational age and black hair color. Epstein's pearl was significantly associated with NVD; on the other hand, erythema toxicum was significantly associated with C/S. There was a significant relationship between milia and increasing gestational age [8].

The prevalence of milia in previous studies has ranged from 1.4 to 93.1% [11–15]. In our study, it was found in 542 (45.2%) of neonates, which is similar to the previous report from Iran [8, 9]. The association of milia with genders has been conflicting across studies. Significant association of male gender has been reported in by Zagne and Fernandes [15] and Behera et al. [16]. On the other hand, Gokdemir et al. [12] and Jain et al. [17] reported a significant association of female gender with milia.

Erythema toxicum was noted in 43% of our population, which is conflicting with both Iranian studies [8, 9]. However, non-Iranian studies reported a lower incidence of erythema toxicum [4, 18]. Positive correlation with C/S and term gestation was reported in previous studies. In our study, it was shown to be significantly more common in males, which is similar to earlier reports from Behera et al. [16].

Salmon patch was the third common cutaneous lesion in our study, with an incidence of 37.3%, which was in the range of 14.5 to 52% reported in previous Iranian studies [7–9].

Mongolian spots are bluish grey macules that evolve in consequence of the residual melanocytes trapped in the dermis in the course of embryonal migration. It affected 37% of our neonates' population that was lower than previous studies in Iran, India, and Brazil [4, 8, 9, 19]. Earlier, Ferahbas et al. [13] and Khoshnevisasl et al. [8] both suggested that the Mongolian spots are related to gestational age and hair color but no association with sex and mode of delivery. Our findings suggested a significant association of Mongolian spots with the male gender.

Skin desquamation was observed in 21.1% of our population, which is relatively similar to the study in Brazil [18]. In contrast to Behera et al., our study found a significant association with female gender and incidence of skin desquamation [16].

Genital hyperpigmentation was the fifth prevalent dermatological finding in our study and was observed in 50 of the male population and 4 of our females with a significantly higher frequency rate of 4.2% and 0.3%, respectively.

We also assessed the associations between neonatal-maternal factors and prevalence and type of cutaneous lesions in neonates during 72 hours after their delivery. Our data suggest that there were no significant correlation between neither maternal age nor the number of pregnancies with an incidence of cutaneous lesions. Interestingly, in the study conducted in the northwest of Iran, maternal age was the only factor that showed a statistically significant association with salmon patch. In another study conducted in Messina, Italy, the skin lesion frequency was significantly higher in those born to primigravida mothers [7, 20]. There was a higher rate of cutaneous lesions among neonates who were born through NVD in comparison with those with C/S in the present study. Mechanical trauma during delivery and higher gestational age among NVD neonates can be the reason for this difference.

This is the first study in the north of Iran to assess associations between dermatological findings and maternal-neonatal factors. The advantage of our study was focusing on maternal-neonatal factors in detail in addition to frequency rates of lesions, while other similar studies in Iran had discussed only prevalence. However, our data are limited to two hospitals located in the west of Mazandaran province. Therefore, we recommend further studies in different hospitals. Also, in our study, we did not measure the association of neonatal-maternal factors with location and type of cutaneous lesions; this matter can be addressed in future studies.

## 5. Conclusion

To the best of our knowledge, our study is the first that investigates the incidence of skin lesions during the first three days of life in neonates from the north of Iran. In this study, 79.8% of newborns had at least one cutaneous lesion. The prevalence of milia, erythema toxicum, salmon patch, and Mongolian spots were 45.2%, 43%, 37.3%, and 37%, respectively. NVD, use of medication, term gestation, and maternal disease were associated with a higher incidence of cutaneous lesions in neonates. Cutaneous lesions are known to be affected by maternal hormones, especially androgens [4]. Consequently, with a longer gestational period, the fetus is more exposed to maternal hormones and more cutaneous lesions develop. Milia, erythema toxicum, Mongolian spots, and genital hyperpigmentation were seen more frequently in the male gender. Conversely, skin desquamation was seen more frequently in females. Among maternal diseases, GDM, UTI, preeclampsia, hypertension, psychiatric disorders, and uterine infection were associated with higher skin lesions. These disorders or their treatments can alter hormonal balance during gestation, resulting in a higher prevalence of neonatal cutaneous lesions. On the other hand, UTI and uterine disease are known to affect neonatal skin through direct infection [4]. From a clinical point of view, it must be emphasized that cutaneous findings are common in newborns and are often a source of parental anxiety and medical concern for inexperienced clinicians. For these reasons, comprehensive knowledge of neonatal cutaneous lesions is crucial for clinicians to avoid unnecessary (and, in some cases, potentially harmful) diagnostic and therapeutic measures. This is important particularly in the circumstances, like low-birth-weight and premature neonates, where medical conditions necessitate special care and iatrogenic complications after treatment are common. This study highlights the high prevalence of different cutaneous lesions in the Iranian population and its associated factors in order to deliver a better understanding of neonatal cutaneous lesions in clinical practice.

## Figures and Tables

**Table 1:** Maternal characteristics and their association with cutaneous lesions.

Maternal factors	Number (%)	Association with skin lesions (*P* value)
Maternal age, mean ± SD	26.7 ± 6.3	>0.05
17–24 years	480 (40)	
25 to 32 years	522 (43.5)	
33 to 40 years	198 (16.5)	
Number of pregnancies		>0.05
1	864 (72)	
2	240 (20)	
3	72 (6)	
>3	24 (2)	
Mode of delivery		<0.01
NVD	960 (80)	
C/S	240 (20)	
Use of medication during pregnancy	240 (20%)	<0.01
Maternal disease	314 (26.3)	<0.01

NVD, natural vaginal delivery. C/S, caesarian section.

**Table 2:** Neonatal characteristics and their association with cutaneous lesions.

Neonatal factors	Number	Percentage	Association with cutaneous lesions (*P* value)
Gender (male)	610	50.9	0.758
Gestational age			<0.01
Term	1058	88.2	
Preterm	142	11.8	
Birth weight			0.476
<2500 gr	120	10	
>2500 gr	1080	90	

**Table 3:** Type of maternal disease and their association with cutaneous lesions.

Type of maternal disease	Number	Percentage	Association with cutaneous lesions (*P* value)
Gestational diabetes mellitus (GDM)	92	7.7	<0.01
Urinary tract infection (UTI)	60	5	<0.01
Preeclampsia	42	3.5	<0.05
Hypertension	34	2.8	<0.05
Psychiatric disorders	12	1	<0.01
Uterine infections	12	1	<0.01
Hypothyroidism	12	1	>0.05
Thrombocytopenia	8	0.66	>0.05
Placental abnormalities	6	0.5	>0.05
Asthma	6	0.5	>0.05
Thalassemia	6	0.5	>0.05
Anemia	6	0.5	>0.05
Other diseases	18	1.5	>0.05
Total	314	26.3	<0.01

**Table 4:** The frequency of cutaneous lesions seen in neonates in the first 72 hours of extrauterine life and their distribution according to gender (*n*) (%).

Skin lesion	Total number (*n* = 958)	Male (*n* = 490)	Female (*n* = 468)	Association with cutaneous lesions (*P* value)
Milia	542 (45.2)	306 (25.5)	236 (19.7)	0.0004
Erythema toxicum	516 (43)	282 (23.5)	234 (19.5)	0.02
Salmon patch	448 (37.3)	240 (20)	208 (17.3)	0.17
Mongolian spots	444 (37)	252 (21)	192 (16)	0.001
Skin desquamation	254 (21.1)	112 (9.3)	142 (11.8)	0.015
Genital hyperpigmentation	54 (4.5)	50 (4.2)	4 (0.3)	<0.01
Hemangiomas	22 (1.8)	6 (1)	5 (0.8)	0.06
Pigmented nevus	12 (1)	4 (0.33)	8 (0.66)	0.25
Miliaria	8 (0.66)	4 (0.33)	4 (0.33)	1
Pustular melanosis	6 (0.5)	4 (0.33)	2 (0.16)	0.68
Total number of lesions	2311	1260	1035	

## Data Availability

The data used to support the findings of this study are available from the corresponding author upon request.
